# Ectopic germinal center and megalin defect in primary Sjogren syndrome with renal Fanconi syndrome

**DOI:** 10.1186/s13075-017-1317-x

**Published:** 2017-06-02

**Authors:** Jing Wang, Yubing Wen, Mengyu Zhou, Xiaoxiao Shi, Lanping Jiang, Mingxi Li, Yang Yu, Xuemei Li, Xuewang Li, Wen Zhang, Andrew L. Lundquist, Limeng Chen

**Affiliations:** 1 Nephrology Department, Chinese Academy of Medical Science, Peking Union Medical College Hospital, Tsing Hua University, Beijing, China; 2 Division of Nephrology, Massachussetts General Hospital, Boston, MA USA; 3 Department of Nephrology, Chinese Academy of Medical Science, Peking Union Medical College Hospital, No 1, Shuaifuyan, Wangfujing St, Beijing, 100730 China

**Keywords:** Primary Sjogren syndrome, Ectopic germinal center, Megalin, Cubilin, IL-17

## Abstract

**Background:**

This study reports the clinical and pathological features of 12 cases of primary Sjogren syndrome (pSS) with renal involvement presenting with proximal tubular dysfunction in a single center, and investigates the possible correlation of ectopic germinal center formation and megalin/cubilin down-expression.

**Method:**

Clinical and pathological records were reviewed. Immunohistochemistry was carried out to detect megalin, cubilin, CD21 and IL-17 expression.

**Results:**

Patients presented with different degrees of proximal renal tubule lesion and decreased estimated glomerular filtration rate (eGFR). Renal biopsy revealed tubulointerstitial nephritis, with tubular epithelial cell degeneration, tubular atrophy, interstitial inflammation and focal fibrosis. Immunohistochemistry revealed decreased expression of megalin and cubilin, two important multiligand protein receptors on the brush border of proximal tubular epithelial cells. IL-17 secreted by Th17 subtype effector T cells was diffusely detected in the renal proximal tubule, with a negative correlation of IL-17 and megalin expression. In addition, ectopic germinal centers characterized by CD21^+^ follicular dendritic cells were present in the renal interstitium. In patients with a decreased eGFR, treatment with 4 weeks of glucocorticoid therapy resulted in an improved eGFR in 75% of patients.

**Conclusion:**

We report 12 cases of pSS characterized by Fanconi syndrome. The decreased megalin and cubilin expression may contribute to the proximal tubular reabsorption defect, possibly secondary to Th17 infiltration and formation of ectopic germinal centers.

## Background

Primary Sjogren syndrome (pSS) is a chronic autoimmune epithelialitis targeting exocrine glands, with possible multisystem involvement [1]. Characteristic pathological changes are focal lymphocytic infiltration around the epithelial ducts and production of autoantibody by hyperactive B cells [2]. Renal involvement is observed in pSS, with both tubular and glomerular abnormalities reported. Tubulointerstitial nephritis (TIN) as a result of periepithelial inflammation is a predominant feature of pSS, often with evidence of a distal renal tubule acidosis (RTA) [3–5]. Fanconi syndrome, the result of proximal tubule epithelial cell (PTEC) injury leading to proximal RTA (type II RTA), hypophosphatemia, hypouricemia, aminoaciduria, glycosuria and urine loss of low molecular weight proteins, is a rare manifestation of pSS. To date, fewer than 20 cases have been reported and the underlying pathogenesis or mechanism remains unclear [6].

Ectopic germinal centers (EGCs), nonlymphoid collections of mature B lymphocytes, have been observed in the labial glands of pSS patients, believed to be the result of chronic inflammation [7]. The presence of CD21^+^ follicular dendritic cells is one of the hallmarks of EGCs [8, 9]. EGCs are suggested to be the site of immune stimulation and have been identified in other autoimmune diseases, such as rheumatoid arthritis and Grave’s disease. Self-reactive T lymphocytes and antibodies contribute to the process of tissue destruction and disease progression [10]. Recent studies suggest that Th17 cells, a subset of CD4^+^ T cells, may directly contribute to lymphoneogenesis in labial glands of pSS patients [11], but it is unclear whether a similar process occurs in the kidney of pSS patients with Fanconi syndrome. Inhibition of receptor-mediated endocytosis has been proposed as the mechanism of Fanconi syndrome in other disease states. Megalin and cubilin are multiligand protein receptors expressed at the brush border membrane and involved in endocytosis in PTECs. Megalin-knockout mice and cubilin-deficient dogs demonstrate deficient endocytosis, reproducing low-molecular proteinuria and vitamin D deficiency, which are the main characteristics of human Fanconi syndrome [12, 13].

In this study, we report the clinical and pathological characteristics and therapeutic outcomes of 12 patients with pSS and Fanconi syndrome. We describe the presence of EGCs in the renal interstitium, the prevalence of Th17/IL-17 expression, and alterations in megalin and cubilin expression, to investigate their possible correlation.

## Methods

### Patients and controls

All patients diagnosed with primary Sjogren syndrome with renal Fanconi syndrome in Peking Union Medical College Hospital (PUMCH) from 1994 to 2014 were enrolled. The diagnosis of pSS was made according to the American–European Consensus Group criteria for pSS [14]. Fanconi syndrome was defined by the coexistence of hypokalemia, hypophosphatemia, normoglycemic glycosuria, generalized aminoaciduria and hyperphosphaturia [15]. Clinical records and follow-up data of enrolled patients were carefully reviewed to understand demographic characteristics, symptoms, physical examination and laboratory tests. Laboratory examinations included routine tests: blood, urine, liver and renal function, 24-h urine protein, erythrocyte sedimentation rate (ESR), C-reactive protein and plasma protein electrophoresis. Renal tubular function assay included: blood and urine electrolytes, blood and urine α_1_-microglobulin (α_1_-MG), β_2_-microglobulin (β_2_-MG), blood pH, carbon dioxide combining power, urine *N*-acetyl-β-amino-glucosidase (NAG), retinol binding protein (RBP) and blood and urine osmotic pressure tests. Immunology assay included: immunoglobulin (IgG, IgA, IgM), rheumatoid factor (RF), blood complement and antinuclear antibodies spectrum. Screening for autoantibodies to SSA/Ro and SSB/La was performed systematically using Ouchterlony double-gel immunodiffusion and western blotting. Other tests included lacrimal and salivary gland secretion test (Schirmer test), salivary scintigraphy, parotid sialography and labial biopsy. The estimated glomerular filtration rate (eGFR) was calculated by the Modification of Diet in Renal Disease (MDRD) study equation [16]. Systemic manifestations of these patients were evaluated by Eular Sjogren’s Syndrome Disease Activity Index (ESSDAI) [17]. Twenty patients with pSS and tubulointerstitial nephritis (pSS + TIN) were included as the control group. TIN clinically manifested as hematuria, leucocyturia, proteinuria (24-h urine protein < 2 g), renal function impairment, distal RTA and hypokalemia, with mainly tubulointerstial impairment in renal biopsies, with or without minor glomerular damage. They had the same workup to exclude proximal tubule injury. Six cases of glomerular minor lesion (GML) were selected as normal controls. These normal patients underwent renal biopsy in the setting of mild isolated hematuria and strong desire for renal biopsy to figure out the etiology, but were not found to have underlying pathology.

### Pathologic studies of kidney tissue

Two-micrometer slides were cut from formalin-fixed and paraffin-embedded (FFPE) sections of kidney tissues, stained with hematoxylin and eosin, periodic acid–Schiff, periodic acid–silver metheramine and Masson trichrome for light microscopy in the laboratory of Nephrology Department at PUMCH. At least eight sections were examined for each patient. All sections were examined by an experienced pathologist who was blinded to the patient’s characteristics. Tubulointerstitial injury was evaluated based on the Oxford Classification of IgA glomerulonephritis [18]. The classification used to describe the degree of lymphocytic infiltration in renal tissue was similar to that used in previous studies of the labial gland in patients with Sjogren syndrome: grade 0 (G0), absent, no lymphocyte infiltration; grade 1 (G1), slight infiltration, scattered lymphocytes infiltrating with an aggregate of fewer than 50 cells; grade 2 (G2), moderate infiltration, focal periductal lymphocytes aggregating in the labial gland, with 50 or more cells per one lesion; and grade 3 (G3), dense infiltration showing EGC-like structures in labial gland and CD21 staining positive [19].

### Immunohistochemistry staining of megalin, cubulin, CD21 and IL-17A

Immunohistochemical (IHC) staining was performed on serial sections using standard methods in five pSS patients with Fanconi syndrome who underwent renal biopsy, five pSS + TIN patients (randomly selected from 20 patients in the control group) and six GML patients. Three-micrometer sections cut from paraffin-embedded tissue were deparaffinized and rehydrated. Sections were heated in a pressure cooker with 0.01 mol/L citrate buffer (pH 6.0) for 5 min to expose antigen and then incubated with the primary antibody (megalin, IL-17A and CD21, rabbit polyclonal antibodies; Abcam, Cambridge, MA, USA; cubilin, goat polyclonal antibody; Santa Cruz, CA, USA) overnight at 4 °C. After incubation with 0.3% H_2_O_2_ for 15 min, sections were incubated with the 1:500 HRP-conjugated anti-rabbit or anti-goat IgG (ImmunoReagents, USA) for 1 h at 37 °C. 3,3′-Diaminobenzidine (DAB) was used as a staining substrate. All section images were captured by a Nikon microscope (Eclipse 80i; Nikon, Japan) equipped with a digital photograph camera (DS-U1; Nikon, Japan).

Megalin immunofluorescence staining was done on 3-μm paraffin sections. The slides were incubated with primary antibody (megalin, rabbit polyclonal antibody; Abcam) overnight at 4 °C. Secondary antibody fluorescein-conjugated AffiniPure donkey anti-rabbit IgG (EarthOx, USA) was applied and incubated at 37 °C for 1 h. The micrographs were taken by confocal laser microscopy (Leica, Germany).

The degree of IHC staining was evaluated by calculating the percentage of positive glomeruli. Staining and scoring were performed blindly on coded slides. At least six fields were selected randomly in the renal cortex of each specimen for photo-documentation. Analytical measurements were done using Image Pro Plus 6.0.

### Statistical analysis

Continuous variables are presented as the mean ± standard deviation. The variables consistent with normal distribution were compared using Student’s *t* test, one-way analysis of variance or Pearson’s correlation coefficients; skewed distribution samples were compared using the Mann–Whitney test or Spearman’s correlation. Categorical variables are expressed as percentages and are compared using the chi-square test. Statistical processing was performed using Graphpad Prism 6.0 and *p* <0.05 was considered statistically significant.

## Results

### Clinical characteristics

From January 1994 to August 2014, 2546 hospitalized patients in PUMCH were diagnosed of pSS, 335 of them presenting with renal manifestation (different degree of haematuria, proteinuria, tubule acidosis or renal function defect). By screening the medical records, 12 (0.47%) pSS patients with Fanconi syndrome were identified, five of whom underwent renal biopsy due to renal function impairment (eGFR compared with those without renal biopsy: 30.0 ± 8.5 vs 98.1 ± 27.5, *p* < 0.001). They were predominantly female, with a mean age of 39.3 ± 8.6 years. The male:female ratio was 1:5. At the onset, six patients presented with fatigue or anoxia, two developed hypokalemia paralysis, one developed bone pain and one had proteinuria. Two patients had sicca syndrome as the chief complaint initially. Systemic manifestations evaluated by ESSDAI ranged from 7 to 27, with an average of 18.8 ± 7.8. Weight loss (58.3%) was the most prominent symptom. Anemia (58.3%, hemoglobin averaged 105.3 ± 25.3 g/L), glandular involvement (16.7%), cutaneous lesion (8.3%) and pulmonary involvement (8.3%) were also observed. ANA was elevated in 75% of patients, and anti-Ro/SSA or anti-La/SSB antibodies were detected in eight (42.7%) and three (25.0%) patients, respectively. Eight of 11 (72.7%) patients showed increased immunoglobulin level. Two of 12 (16.7%) patients had increased RF level, and 3/12 (25%) patients showed decreased blood complement level. Laboratory studies consistent with proximal tubular injury were common with hypokalemia (100%), hypophosphatemia (83.3%), glycosuria (83.3%), RTA (75.0%) and aminoaciduria (72.7%). It is noteworthy that a majority of patients were associated with defects in other parts of the tubule, as proximal and distal RTA always coexistent. Eleven of 12 patients presented with albuminuria, ranging from trace to 1.0 g/L. Increased total protein in the urine was elevated in 10 patients, averaging 1.8 ± 0.5 g/24 h. Average eGFR was 69.8 ± 40.9 ml/min/1.73 m^2^, with five patients (41.7%) under 60 ml/min/1.73 m^2^. Table 1 compares the clinical profile between pSS patients with Fanconi syndrome and those with tubulointerstitial nephritis (pSS + TIN) who did not present with proximal tubule dysfunction. The pSS + Fanconi group had lower serum phosphorus level and more prominent proteinuria. Systemic manifestations showed no significant difference.

**Table 1:** Comparison of clinical profile between patients with pSS + Fanconi syndrome and pSS + TIN

	pSS with Fanconi syndrome	pSS with TIN	*P* value*
	(*N* = 12)	(*N* = 20)	
Age (years)	39.3 ± 8.6	37.6 ± 12.2	0.366
Gender (female %)	10 (83.3)	18 (90.0)	0.620
Presenting symptoms			
Polyuria	8 (66.7)	10 (50.0)	0.471
Muscle weakness	9 (75.0)	14 (70.0)	1.000
Nocturia	7 (58.3)	5 (25.0)	0.130
dRTA	9 (75.0)	11 (55.0)	0.452
Paralysis	3 (25.0)	6 (30.0)	1.000
SCr elevation	9 (75.0)	12 (60.0)	0.465
eGFR (ml/min/1.73 m^2^)	66.08 ± 38.20	76.14 ± 39.57	0.716
Serum potassium (mmol/L)	2.79 ± 0.14	2.76 ± 0.24	0.120
Serum calcium (mmol/L)	2.23 ± 0.14	2.13 ± 0.09	0.104
Serum phosphorus (mmol/L)	0.69 ± 0.33	1.02 ± 0.29	0.001
24-h urine protein (g/24 h)	1.51 ± 0.84	1.00 ± 0.61	0.019
Systemic manifestations			
ESSDAI	18.83 ± 7.83	15.50 ± 6.53	0.289
Weight loss (>5%)	7 (58.3)	6 (30.0)	0.150
Glandular	2 (16.7)	3 (15.0)	1.000
Cutaneous	1 (8.3)	1 (5.0)	1.000
Pulmonary	1 (8.3)	3 (15.0)	1.000
Lymphadenopathy	0 (0.0)	2 (10.0)	0.516
HGB (g/L)	105.3 ± 25.2	113.4 ± 13.6	0.408
Immune profile			
ESR (mm/h)	58.8 ± 30.9	42.7 ± 30.9	0.141
ANA positive	9 (75.0)	18 (90.0)	0.338
SSA positive	5 (41.7)	14 (73.7)	0.130
SSB positive	3 (25.0)	19 (47.1)	0.273
IgG (g/L)	18.3 ± 8.2	19.7 ± 6.3	0.588

Data presented as number (%) or mean ± SD unless otherwise noted

*ANA* antinuclear antibody, *dRTA* distal renal tubule acidosis, *eGFR* estimated glomerular filtration rate, *ESR* erythrocyte sedimentation rate, *ESSDAI* Eular Sjogren’s Syndrome Disease Activity Index, *HGB* hemoglobin, *IgG* immunoglobulin G, *pSS* primary Sjogren syndrome, *SCr* serum creatinine, *SSA* Sjogren-syndrome-related antigen A, *SSB* Sjogren-syndrome-related antigen B, *TIN* tubulointerstitial nephritis

*Mann–Whitney (two-tailed) test for continuous variables and Fisher’s exact (two-tailed) test for categorical variables

### Histopathological features

**Fig. 1:**
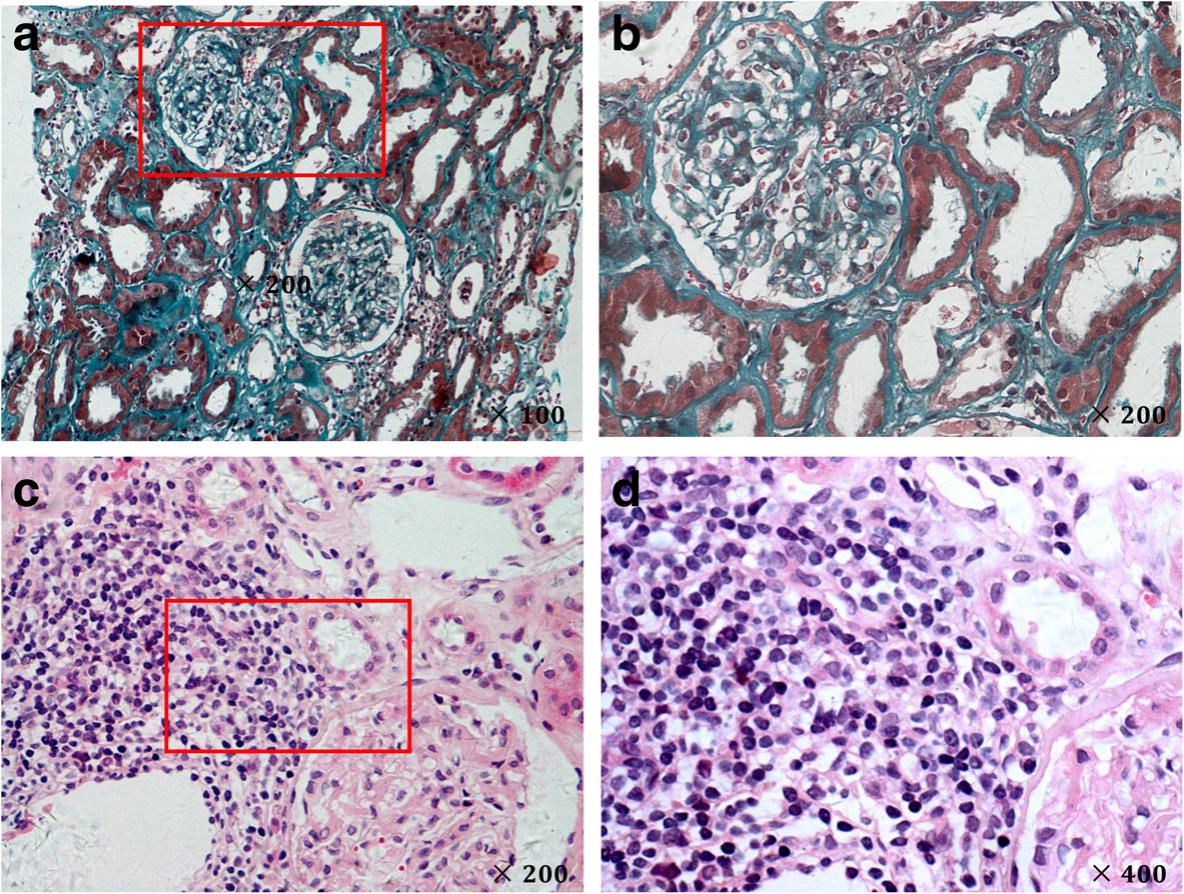
Patients with pSS + Fanconi syndrome show remarkable pathological lesions of renal proximal tubule. **a** Masson staining reveals interstitial fibrosis. **b** Boxed area in **a** enlarged. **c** Hematoxylin and eosin E staining indicating focus of lymphocyte infiltration. **d** Boxed area in **c** enlarged, indicating plasma cells in lymphocytes focus

The biopsies of five pSS patients with Fanconi syndrome who underwent renal biopsy were reviewed. The primary lesion was moderate TIN with minimal glomerular injury and notable proximal tubular injury. Changes included tubule atrophy with defective brush border (64.0 ± 12.1%) and interstitial fibrosis (58.0 ± 12.8%) (Fig. 1a, b). Renal biopsy of Patient 3 revealed acute interstitial nephritis (AIN) with lymphocytic infiltration. Dense lymphocytes, monocytes, eosinophils and plasma cells could be identified in the focal lesion (Fig. 1c, d).

### Megalin and cubilin expression

**Fig. 2:**
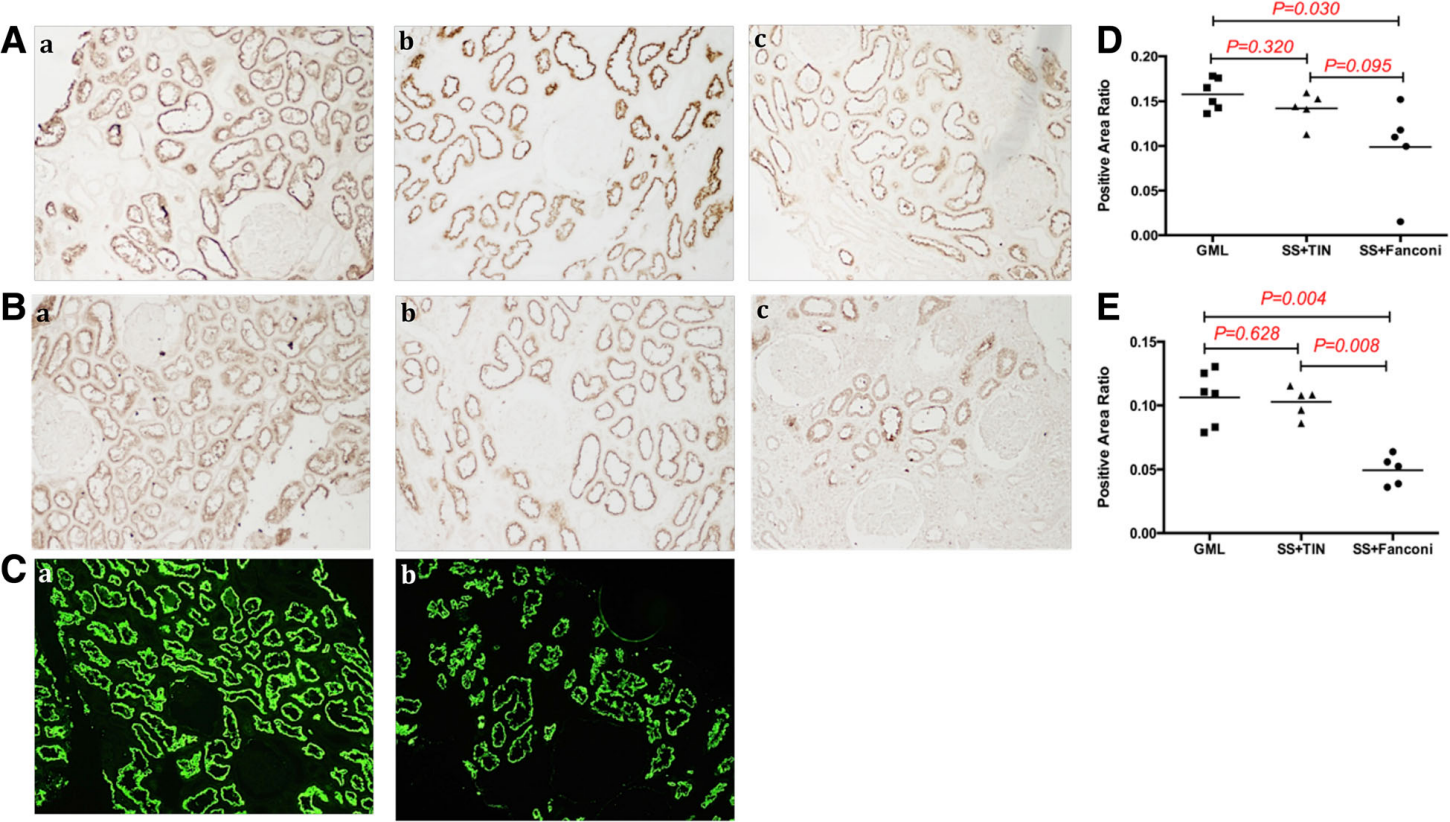
Immunohistochemistry and immunofluorescence reveals loss of megalin and cubilin expression in pSS patients with Fanconi syndrome. Staining of megalin (**A**, **C**) and cubilin (**B**) on kidney biopsies of patients with glomerular minor lesion (GML) (*Aa*, *Ba*, *Ca*), patients of pSS with tubulointerstitial nephritis (*Ab*, *Bb*), and pSS patients with Fanconi syndrome (*Ac*, *Bc*, *Cb*). Semiquantitative analysis shows decreased expression of megalin (**D**) and cubilin (**E**) in pSS patients with Fanconi syndrome, compared with GML and SS + TIN groups. *GML* glomerular minor lesion, *SS* Sjogren syndrome, *TIN* tubulointerstitial nephritis

Compared with normal controls (GML), the expression of megalin and cubilin on proximal tubule cells was decreased (Fig. 2). As shown in semiquantitative analyses, the positive area staining ratio of megalin and cubilin in pSS patients with Fanconi syndrome was significantly lower than that in the control GML group (Fig. 2D, E) (0.10 ± 0.01 vs 0.16 ± 0.02, *p* = 0.030; 0.05 ± 0.01 vs 0.11 ± 0.01, *p* = 0.004, respectively). Megalin and cubilin expression showed no significant difference between pSS + TIN patients and the GML group (0.14 ± 0.01 vs 0.16 ± 0.01, *p* = 0.320; 0.10 ± 0.01 vs 0.11 ± 0.01, *p* = 0.628), while there was a trend toward reduced expression in SS + Fanconi syndrome patients compared with the pSS + TIN group (0.10 ± 0.02 vs 0.14 ± 0.01, *p* = 0.095; 0.05 ± 0.01 vs 0.10 ± 0.01, *p* = 0.008).

### Presence of CD21^+^ germinal centers

**Fig. 3:**
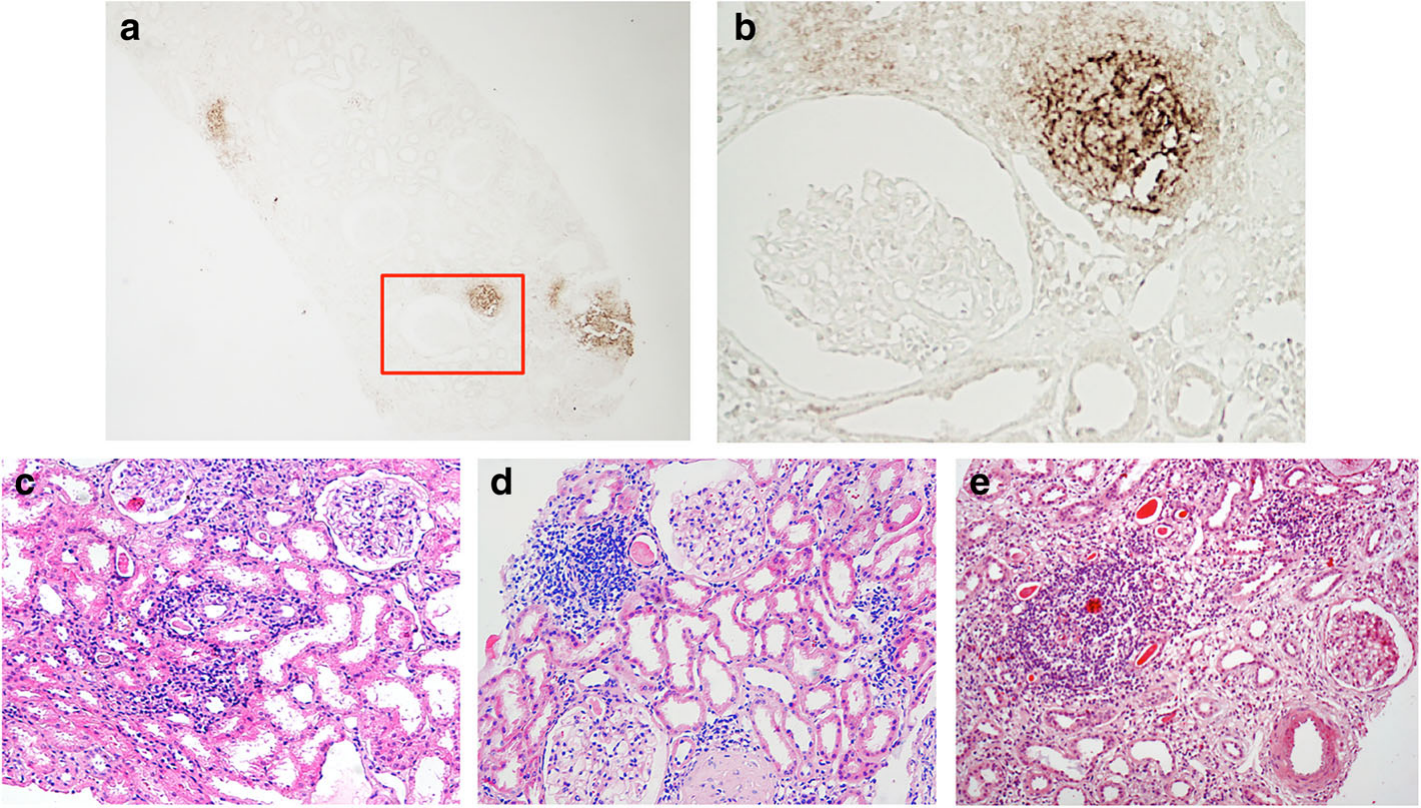
EGC of pSS with Fanconi syndrome. Immunohistochemical staining for CD21 in the kidney cortex (**a**, **b**) (boxed area in **a** is enlarged in **b**: **a** × 40, **b** × 200). Classification of lymphocytes aggregating in the proximal tubule: **c** G1, **d** G2 and **e** G3

Focuses of lymphocytic infiltration could be seen in all biopsies of patients with pSS and Fanconi syndrome. There were three of G1, two of G2 and one of G3. The dendritic cell marker CD21^+^ was observed in one patient who showed the characteristics of AIN. There were several CD21^+^ focuses, indicating the presence of EGCs scattered in the renal interstitium (Fig. 3). We observed that the patient with G3 lymphocyte focus showed the lowest megalin and cubilin positive staining ratio. No specific relationship was identified between focus grading and expression levels of megalin or cubilin due to the small sample size.

### Expression of IL-17A and megalin in serial sections

**Fig. 4:**
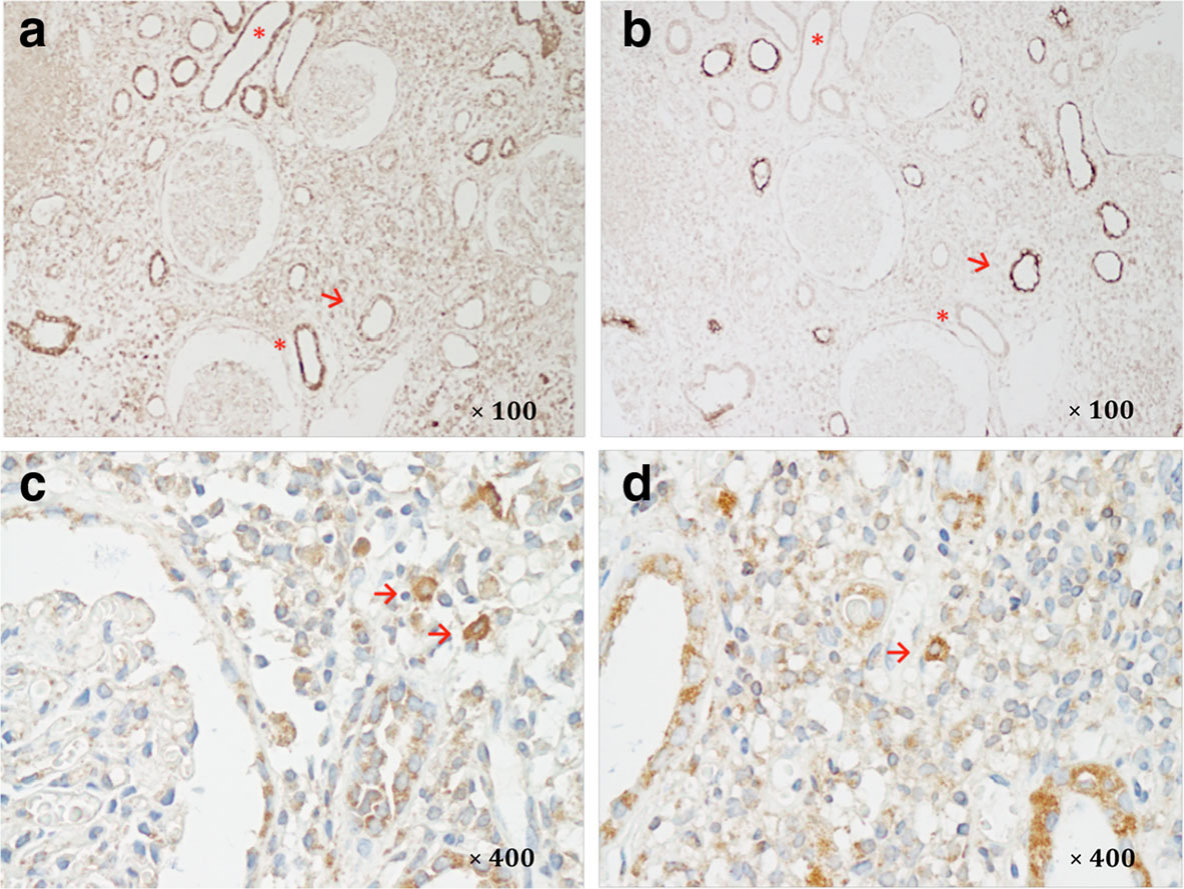
Expression of IL-17A and megalin in serial sections. Immunohistochemical staining of IL-17A (**a**) and megalin (**b**) on adjacent kidney paraffin sections from a patient with pSS + Fanconi syndrome. Proximal tubules with high expression of IL-17A show loss of megalin (*asterisks*), and those with preserved megalin expression have no IL-17A staining (*arrow*). **c**, **d** Identification of IL-17A expressing cells (*arrow*) in renal interstitium with hematoxylin staining of nucleus

Diffuse expression of IL-17, a proinflammatory cytokine contributing to the formation of germinal centers, was observed in proximal renal tubules with variable intensity, while none was observed in the glomerulus. Both cytoplasm and cell membranes of the proximal renal tubule stained positively. It was noticeable that in the adjacent kidney paraffin sections of Patient 3, proximal tubules with strong IL-17A staining lacked expression of megalin, and vice versa (Fig. 4a, b). Infiltrating cells expressing IL-17A were identified in renal interstitium after hematoxylin staining of nucleus (Fig. 4c, d).

### Treatment and follow-up

All patients received treatment primarily with glucocorticosteroids with an initial dose of prednisone typically 0.5–1 mg/kg/day. Three patients also received cyclophosphamide or methotrexate in addition to prednisone. Patients generally showed good response to treatment, with a significant increase of eGFR (26–235%) over the median in-hospital time of 3 weeks. Five patients with impaired renal function were followed for an average of 59 months (1–192 months). During the follow-up, all patients showed improved renal function, with SCr decreased 39.2% on average. Their renal function remained stable with no flares (SCr higher than at onset, with/or 24-h UP > 0.3 g) and no requirement for renal replacement therapy. Electrolyte derangement also corrected with treatment, allowing a reduction in oral supplementation.

## Discussion

The most common renal manifestation of pSS is a distal RTA, with proximal tubular acidosis seldom reported [20]. Fanconi syndrome, general dysfunction of the proximal tubule, is a relatively rare clinical manifestation of pSS. By searching PubMed with ((Sjogren syndrome) or (autoimmune epithelialitis)) and ((renal tubule) OR Fanconi), about 20 cases were identified [6, 21–35]. Wang et al. [6] and Shi and Chen [33] have summarized SS-related Fanconi syndrome cases that had been reported. We made a supplement to their summarization. We reported 12 cases of pSS + Fanconi syndrome cases with detailed clinical profile and follow-up records, to our limited knowledge, which is the largest sample in a single center. As presented in Table 2, similar clinical and pathological characteristics were observed in our study. In our series, 58% patients had impaired renal function with moderate TIN, and showed good response to glucocorticoid therapy. Both tubular function injury and eGFR were improved following treatment with steroids.

**Table 2:** Comparison of clinical profile between our cases and pSS-related Fanconi syndrome reported in the literature

Reference	Presenting symptoms	RTA	Cr	K (mmol/L)	Histology	Treatment	Outcome
Shearn and Tu [29]	Polyuria	+	n.a.	3.8	TIN, tubular atrophy	n.a.	n.a.
Walker et al. [30]	Paralysis, polyuria	+	n.a.	n.a.	TIN	Prednisolone 10 mg/day	n.a.
Kamm and Fischer [24]	Polyuria, nocturia, weight loss	+	2.7 mg/dl	2.9	Diffuse TIN	Supportive only	Improved
Matsumura et al. [26]	n.a.	n.a.	2.7 mg/dl	n.a.	TIN, tubulitis	n.a.	n.a.
Ardiles et al. [21]	Muscle weakness	+	1.3 mg/dl	2.5	n.a.	Prednisolone “low dose”	Improved
Bridoux et al. [22]	Weight loss	+	1.8 mg/dl	3.5	Diffuse TIN, proximal tubulitis	Supportive only	Died^a^
	Polyuria	+	1.6 mg/dl	2.4	Diffuse TIN, proximal tubulitis	Prednisolone 10 mg/day	Improved
Kobayashi et al. [25]	Muscle weakness	+	1.3 mg/dl	2.7	Diffuse TIN, proximal tubule atrophy	Prednisolone 30 mg/day, 6 months later 12.5 mg/day	Improved
Ren et al. [32]^b^	n.a.						
Yang et al. [31]	Muscle weakness, respiratory distress	+	1.4 mg/dl	2.7	n.a.	Supportive only	n.a.
Nakamura et al. [27]	Renal dysfunction, organizing pneumonia, multiple bone fracture	+	1.3 mg/dl	3.0	n.a.	Mizoribine 50 mg/day	n.a.
Wang et al. [6]	Hypokalemic paralysis	+	2.2 mg/dl	1.6	Diffuse TIN	Mycophenolate mofetil 1 g/day	Improved^f^
Ram et al. [28]	Paralysis	+	2.1 mg/dl	1.3	Dense lymphocytic interstitial infiltrate	Supportive only	Improved
Celik et al. [23]	Paralysis, cardiac arrest^c^	+	1.1 mg/dl	1.1	n.a.	Prednisone 40 mg/d iv. in acute phase	Improved^f^
Shi and Chen [33]	Proteinuria, glycosuria^d^	+		3.07	TIN	Methylprednisolone	Improved^e^
Saeki et al. [34]	Renal dysfunction	+	1.07 mg/dl	3.7	TIN	Prednisolone 40 mg/day	Improved^e^
Kong et al. [35]	Weakness, osteodynia, impaired mobility	_	n.a.	1.3	n.a.	Prednisone 30 mg/day	
Our cases	Fatigue, anorexia	+	151 μmol/L	3.4	Diffuse TIN, diffuse tubule atrophy, lymphocyte infiltration	Prednisone 50 mg/day	Improved^f^
	Fatigue, polyuria, anorexia, osteopathy	+	88 μmol/L	2.1	Focal TIN, focal tubule atrophy	Prednisone 40 mg/day	Improved^f^
	Fatigue, anorexia, osteopathy	+	176 μmol/L	3.3	Diffuse TIN, diffuse tubule atrophy, lymphocyte infiltration	Prednisone 50 mg/day	Improved^e^
	Fatigue, anorexia	+	305 μmol/L	2.7	Focal TIN, focal tubule atrophy	Prednisone 45 mg/day	Improved^e^
	Fatigue, anorexia, polyuria	–	72 μmol/L	2.53	Mild tubulitis	Supportive only	Improved^f^
	Fatigue, anorexia, polyuria	+	184 μmol/L	3.0	n.a.	Prednisone 35 mg + cyclophosphamide 0.2 g qod	Improved^e^
	Polyuria	–	120 μmol/L	3.4	n.a.	Prednisone 60 mg/day	Improved^e^
	Fatigue, polyuria, anorexia, osteopathy	+	202 μmol/L	2.62	n.a.	Prednisone 55 mg/day	Improved^e^
	Osteopathy	–	110 μmol/L	3.2	n.a.	Prednisone 55 mg/day + metrotraxate 10 mg qw	Improved^f^
	Fatigue, polyuria, osteopathy	+	75 μmol/L	2.88	n.a.	Prednisone 40 mg/day + cyclophosphamide 0.2 g qod	Improved^f^
	Hypokalemic paralysis, osteopathy	+	65 μmol/L	2.1	n.a.	Supportive	Improved^f^
	polyuria, osteopathy	+	71 μmol/L	2.34	n.a.	Prednisone 30 mg/day + metrotraxate 10 mg qw	Improved^f^

Mean age of patients with pSS-related Fanconi syndrome reported in the literature is 47.4 ± 13.2, with a female ratio of 93.3%

*n.a.* not available, *pSS* primary Sjogren syndrome, *qop* every other day, *qw* every week, *RTA* renal tubule acidosis, *TIN* tubulointerstitial nephritis

^a^Probable cardiovascular event

^b^Four cases reported in a retrospective study of 130 cases, no detailed information

^c^Also diagnosed of brucellic disease

^d^With autoimmune thyroiditis

^e^Improvement of renal function, and stable during follow-up

^f^Correction of electrolyte derangement, and relief of symptoms

Few studies have focused on the mechanism of Fanconi syndrome or PTEC injury in pSS. We observed the downregulation of megalin and cubilin in PTECs, which suggests defective endocytosis. Megalin and cubilin are endocytic receptors coexpressed in the proximal tubule, located on the brush border and endocytic vesicles. They bind and mediate the endocytosis of a variety of ligands, including enzymes or enzyme inhibitors, lipoproteins, hormones, signaling proteins, immune or stress response-related proteins, receptors and vitamin carrier proteins as well as drugs and toxins [12, 36]. Megalin and cubilin are associated with key processes to fulfill the classic reabsorption function of proximal tubules: the integrity of cell structure, such as polarity, brush border and endocytic apparatus; apical multiligand receptors megalin and cubilin; and intact transport system consisting of microvilli, clathrin-coated pits, early endosomes, late endosomes and lysosome. The other processes include energy production by mitochondria and basolateral Na^+^-K^+^-ATPase as the driving force for Na^+^-coupled transport [13, 37–39]. In cystinosis, a known cause of Fanconi syndrome, alterations in megalin activity have been noted on the brush border, endosomes and lysosomes by immunofluorescence under electron microscopy [40]. Megalin and cubilin have also been shown to be critical in Fanconi syndrome from other causes. Mutations in the gene low-density lipoprotein receptor-related protein 2 (*LRP2*), encoding the protein megalin, have been identified in Donnai-Barrow (DB) syndrome and Facio-Oculo-Acustico-Renal (FOAR) syndrome. These patients show prominent low-molecular weight proteinuria, with malabsorption of vitamin D-binding protein, retinol-binding protein and albumin [41]. Elegant experiments have suggested urinary megalin deficiency implicating abnormal tubular endocytic function in Fanconi syndrome related to Dent’s disease and Lowes syndrome [42]. In these genetic disorders, the down-expression of megalin/cubilin caused by impaired endosome–lysosome trafficking has also been shown [43, 44]. On the other hand, megalin-mediated endocytosis of excessive protein is pathogenic in light-chain tubulopathy. It has been shown that silencing megalin and cubilin genes may inhibit myeloma light chain uptake, suppressing inflammation in PTECs, and reducing the nephrotoxic effects [45]. The mechanism of drug-induced Fanconi syndrome is not fully understood at this point in time [46]. However, as far as we can see, the mechanism of Fanconi syndrome caused by pSS has not yet been discussed. The innovation of our study is to show that defect endocytosis in PTECs mediated by megalin and cubilin may contribute to the reabsorption impairment in patients with pSS and Fanconi syndrome. A trend toward loss of megalin expression was observed in pSS with TIN, but this was not significant compared with the decreased expression of megalin noted in patients with pSS and Fanconi syndrome. We propose that proximal tubule impairment was less severe in TIN with general tubule injury, because no symptoms of endocytic receptor defect were observed.

The mechanism of megalin and cubilin alteration remains poorly understood, although past studies have indicated that renal ischemia and reperfusion injury, inflammation and drugs may all be causative. Lipopolysaccharide has been shown to downregulate megalin and cubulin expression in vitro and vivo [47]. In this study, we first observed the inverse relationship of megalin and IL-17 expression with associated ectopic germinal center (EGC) formation in the kidney. IL-17 is a proinflammatory cytokine secreted by Th17 cells, which comprise a distinct subset of CD4^+^ T cells that play a role in autoimmune disease [48]. Th17 cells are believed to activate follicular dendritic cells and stromal cells via surface molecule LTα1β2 and they secrete IL-17, stimulating stromal cells, fibroblasts and tissue epithelial cells to produce chemokines [11]. IL-17 secretion is induced by IL-21, produced by Tfh cells, a component of EGCs. IL-17 then acts in conjunction with IFNγ to recruit lymphocytes for the formation of the germinal center [49]. In addition, we observed that renal proximal tubule cells can express IL-17. It is reasonable to assume that epithelial cells may be an important component of immune response in TIN, as has been indicated in renal transplant rejection. EGCs are highly organized lymphoid aggregates that form in tissue sites which are not typically associated with lymphoid neogenesis [50]. TIN is characterized by organized infiltration of anatomically distinct and adjacent T-cell and B-cell compartments, with the presence of follicular dendritic cells (DCs) [9]. As the infiltrating cells, DCs have been shown to be necessary and sufficient in the formation of EGCs [51–53], Depletion of DCs leads to disappearance of existing follicular germinal center structure [52]. We observed that circulating pDCs and mDCs were reduced in pSS patients with TIN, compared with patients with pSS alone. In addition, immunohistochemical staining of BDCA-2 and DC-SIGN revealed increased pDCs and mDCs in renal interstitium. Combining these findings, we assume that in the setting of local inflammation in pSS, DCs can be recruited to renal tissue from peripheral blood (unpublished data). CD21 remains the most reliable marker of follicular dendritic cells. In another study of our group, we find that in pSS with membranous nephrology, all G3 patients (30.5% of 36 patients) showed positive CD21 staining with varying degree, and typical EGC structure was seen in nine patients. The relationship of IL-17 and germinal center activity has been observed previously. In an experimental autoimmune encephalomyelitis murine model, Th17 cells also directly elicit formation of ectopic lymphoid follicle formation, by surface molecule podoplanin and secretion of IL-17 [54]. In the formation of inducible bronchus associated lymphoid tissue (iBALT), IL-17 secreted by Th17 cells contributes to the initial stage [55]. In the labial glands with pSS, germinal centers developed in 25.1 ± 5.0% of patients [56]. Both Sakai et al. and Fei et al. reported that the majority infiltrating cells in the salivary glands of SS patients were CD4^+^ T cells, with a predominant expression of IL-17 [56, 57], which could be modified by immunosuppressive treatment [57]. Consistent with their findings, we observed infiltrating cells expressing IL-17A present in renal interstitium (we did not perform CD4 and IL-17A double-staining to clarify Th17 cells due to limited pathological sections). There is also evidence to suggest that EGC formation may contribute to progression of disease in lupus nephritis [58, 59]. Therefore, the presence of DCs and EGCs in the interstitium of the kidney in patients with pSS suggests that the severe renal interstitial inflammation, with Th17 infiltration and IL-17 secretion, may be correlated to megalin and cubilin impairment. We suppose that local inflammation may subsequently mediate brush border destruction, characterized by decreased megalin and cubilin expression, leading to the reabsorptive dysfunction of PTECs and Fanconi syndrome.

There are several limitations of our study. First, not all patients underwent bicarbonate loading test, and patients with isolated proximal tubule acidosis could not be identified. Second, as a retrospective study, instead of the mechanism we only suggested a correlation between EGC formation and defected endocytosis characterized by megalin/cubilin down-expression. It is possible that megalin/cubilin deficiency is a reflection of generalized proximal tubule injury, because up to 45.2% of pSS patients were present with elevated excretion of β_2_-microglobulin. Third, a different degree of inflammation may not explain the whole picture of why only certain pSS + TIN patients present with proximal tubule defect. Whether this is due to specific local antibody remains to be elucidated by further study.

## Conclusion

Collectively, we reported 12 cases of pSS with Fanconi syndrome. They present with proximal tubular defect, decreased eGFR and tubulointerstitial nephritis, and they show good response to glucocorticosteroids therapy. Down-expression of megalin and cubilin may cause the endocytosis reabsorption dysfunction of proximal renal tubules. We suggest that this is related to Th17 infiltration, IL-17 expression and formation of ectopic germinal centers.

## Abbreviations

ANA: Antinuclear antibody
DC: Dendritic cell
EGC: Ectopic germinal center
eGFR: Estimated glomerular filtration rate
ESR: Erythrocyte sedimentation rate
ESSDAI: Eular Sjogren’s Syndrome Disease Activity Index
FFPE: Formalin-fixed and paraffin-embedded
GML: Glomerular minimal lesion
IL-17: Interleukin 17
MDRD: Modification of Diet in Renal Disease
NAG: *N*-acetyl-β-amino-glucosidase
pSS: Primary Sjogren syndrome
PTEC: Proximal tubule epithelial cell
RBP: Retinol binding protein
RTA: Renal tubule acidosis
SCr: Serum creatinine
SSA: Sjogren-syndrome-related antigen A
SSB: Sjogren-syndrome-related antigen B
TIN: Tubulointerstitial nephritis
α_1_-MG: α_1_-Microglobulin
β_2_-MG: β_2_-Microglobulin

## References

[1] Moutsopoulos HM (1994). Sjogren's syndrome: autoimmune epithelitis. Clin Immunol Immunopathol.

[2] Ramos-Casals M, Tzioufas AG, Font J (2005). Primary Sjogren's syndrome: new clinical and therapeutic concepts. Ann Rheum Dis.

[3] Maripuri S, Grande JP, Osborn TG, Fervenza FC, Matteson EL, Donadio JV (2009). Renal involvement in primary Sjogren's syndrome: a clinicopathologic study. Clin J Am Soc Nephrol.

[4] Goules AV, Tatouli IP, Moutsopoulos HM, Tzioufas AG (2013). Clinically significant renal involvement in primary Sjogren's syndrome: clinical presentation and outcome. Arthritis Rheum.

[5] Kaufman I, Schwartz D, Caspi D, Paran D (2008). Sjogren's syndrome—not just Sicca: renal involvement in Sjogren's syndrome. Scand J Rheumatol.

[6] Wang CC, Shiang JC, Huang WT, Lin SH (2010). Hypokalemic paralysis as primary presentation of Fanconi syndrome associated with Sjogren syndrome. J Clin Rheumatol.

[7] Theander E, Vasaitis L, Baecklund E, Nordmark G, Warfvinge G, Liedholm R (2011). Lymphoid organisation in labial salivary gland biopsies is a possible predictor for the development of malignant lymphoma in primary Sjogren's syndrome. Ann Rheum Dis.

[8] Risselada AP, Looije MF, Kruize AA, Bijlsma JW, van Roon JA (2013). The role of ectopic germinal centers in the immunopathology of primary Sjogren's syndrome: a systematic review. Semin Arthritis Rheum.

[9] Neyt K, Perros F, GeurtsvanKessel CH, Hammad H, Lambrecht BN (2012). Tertiary lymphoid organs in infection and autoimmunity. Trends Immunol.

[10] Aloisi F, Pujol-Borrell R (2006). Lymphoid neogenesis in chronic inflammatory diseases. Nat Rev Immunol.

[11] Grogan JL, Ouyang W (2012). A role for Th17 cells in the regulation of tertiary lymphoid follicles. Eur J Immunol.

[12] Christensen EI, Birn H (2002). Megalin and cubilin: multifunctional endocytic receptors. Nat Rev Mol Cell Biol.

[13] Sirac C, Bridoux F, Essig M, Devuyst O, Touchard G, Cogne M (2011). Toward understanding renal Fanconi syndrome: step by step advances through experimental models. Contrib Nephrol.

[14] Vitali C, Bombardieri S, Jonsson R, Moutsopoulos HM, Alexander EL, Carsons SE (2002). Classification criteria for Sjogren's syndrome: a revised version of the European criteria proposed by the American–European Consensus Group. Ann Rheum Dis.

[15] Roth KS, Foreman JW, Segal S (1981). The Fanconi syndrome and mechanisms of tubular transport dysfunction. Kidney Int.

[16] Levey AS, Bosch JP, Lewis JB, Greene T, Rogers N, Roth D (1999). A more accurate method to estimate glomerular filtration rate from serum creatinine: a new prediction equation. Modification of Diet in Renal Disease Study Group. Ann Intern Med.

[17] Seror R, Ravaud P, Bowman SJ, Baron G, Tzioufas A, Theander E (2010). EULAR Sjogren's syndrome disease activity index: development of a consensus systemic disease activity index for primary Sjogren's syndrome. Ann Rheum Dis.

[18] Roberts IS, Cook HT, Troyanov S, Alpers CE, Amore A, Barratt J (2009). The Oxford classification of IgA nephropathy: pathology definitions, correlations, and reproducibility. Kidney Int.

[19] Ohara T, Itoh Y, Itoh K (2000). Reevaluation of laboratory parameters in relation to histological findings in primary and secondary Sjogren's syndrome. Intern Med.

[20] Kronbichler A, Mayer G (2013). Renal involvement in autoimmune connective tissue diseases. BMC Med.

[21] Ardiles L, Ramirez P, Calderon S, Aguirre V, Poblete MT (2001). Life-threatening hypokalemic paralysis and hypophosphatemic myopathy as initial presentations of primary Sjogren's syndrome. J Clin Rheumatol.

[22] Bridoux F, Kyndt X, Abou-Ayache R, Mougenot B, Baillet S, Bauwens M (2004). Proximal tubular dysfunction in primary Sjogren's syndrome: a clinicopathological study of 2 cases. Clin Nephrol.

[23] Celik G, Ozturk E, Ipekci SH, Yilmaz S, Colkesen F, Baldane S (2014). An uncommon presentation of Sjogren's syndrome and brucellosis. Transfus Apher Sci.

[24] Kamm DE, Fischer MS (1972). Proximal renal tubular acidosis and the Fanconi syndrome in a patient with hypergammaglobulinemia. Nephron.

[25] Kobayashi T, Muto S, Nemoto J, Miyata Y, Ishiharajima S, Hironaka M (2006). Fanconi's syndrome and distal (type 1) renal tubular acidosis in a patient with primary Sjogren's syndrome with monoclonal gammopathy of undetermined significance. Clin Nephrol.

[26] Matsumura R, Kondo Y, Sugiyama T, Sueishi M, Koike T, Takabayashi K (1988). Immunohistochemical identification of infiltrating mononuclear cells in tubulointerstitial nephritis associated with Sjogren's syndrome. Clin Nephrol.

[27] Nakamura H, Kita J, Kawakami A, Yamasaki S, Ida H, Sakamoto N (2009). Multiple bone fracture due to Fanconi's syndrome in primary Sjogren's syndrome complicated with organizing pneumonia. Rheumatol Int.

[28] Ram R, Swarnalatha G, Ashok KK, Madhuri HR, Dakshinamurty KV (2012). Fanconi syndrome following honeybee stings. Int Urol Nephrol.

[29] Shearn MA, Tu WH (1965). Nephrogenic diabetic insipidus and other defects of renal tubular function in Sjoergren's syndrome. Am J Med.

[30] Walker BR, Alexander F, Tannenbaum PJ (1971). Fanconi syndrome with renal tubular acidosis and light chain proteinuria. Nephron.

[31] Yang YS, Peng CH, Sia SK, Huang CN (2007). Acquired hypophosphatemia osteomalacia associated with Fanconi's syndrome in Sjogren's syndrome. Rheumatol Int.

[32] Ren H, Wang WM, Chen XN, Zhang W, Pan XX, Wang XL (2008). Renal involvement and followup of 130 patients with primary Sjogren's syndrome. J Rheumatol.

[33] Shi M, Chen L. Sjogren's syndrome complicated with Fanconi syndrome and Hashimoto's thyroiditis: case report and literature review. J Int Med Res. 2016;44(3): 753-9. 10.1177/0300060515593767 PMC5536691 26966155

[34] Saeki T, Nakajima A, Ito T, Takata T, Imai N, Yoshita K, et al. Tubulointerstitial nephritis and Fanconi syndrome in a patient with primary Sjogren's syndrome accompanied by antimitochondrial antibodies: a case report and review of the literature. Mod Rheumatol. 2016;4:1–4. (Epubahead of print). 10.3109/14397595.2016.1174422 27142563

[35] Kong DH, Gao H, Qiu MC (2005). Sjogren's syndrome, renal tubular acidosis and Fanconi syndrome—a case report. Zhonghua Yi Xue Za Zhi.

[36] Christensen EI, Birn H, Storm T, Weyer K, Nielsen R (2012). Endocytic receptors in the renal proximal tubule. Physiology (Bethesda).

[37] Gburek J, Verroust PJ, Willnow TE, Fyfe JC, Nowacki W, Jacobsen C (2002). Megalin and cubilin are endocytic receptors involved in renal clearance of hemoglobin. J Am Soc Nephrol.

[38] Kozyraki R, Fyfe J, Verroust PJ, Jacobsen C, Dautry-Varsat A, Gburek J (2001). Megalin-dependent cubilin-mediated endocytosis is a major pathway for the apical uptake of transferrin in polarized epithelia. Proc Natl Acad Sci U S A.

[39] Leheste JR, Rolinski B, Vorum H, Hilpert J, Nykjaer A, Jacobsen C (1999). Megalin knockout mice as an animal model of low molecular weight proteinuria. Am J Pathol.

[40] Gaide Chevronnay HP, Janssens V, Van Der Smissen P, N'Kuli F, Nevo N, Guiot Y (2014). Time course of pathogenic and adaptation mechanisms in cystinotic mouse kidneys. J Am Soc Nephrol.

[41] Pober BR, Longoni M, Noonan KM (2009). A review of Donnai-Barrow and facio-oculo-acoustico-renal (DB/FOAR) syndrome: clinical features and differential diagnosis. Birth Defects Res A Clin Mol Teratol.

[42] Norden AG, Lapsley M, Igarashi T, Kelleher CL, Lee PJ, Matsuyama T (2002). Urinary megalin deficiency implicates abnormal tubular endocytic function in Fanconi syndrome. J Am Soc Nephrol.

[43] Santo Y, Hirai H, Shima M, Yamagata M, Michigami T, Nakajima S (2004). Examination of megalin in renal tubular epithelium from patients with Dent disease. Pediatr Nephrol.

[44] Wilmer MJ, Emma F, Levtchenko EN (2010). The pathogenesis of cystinosis: mechanisms beyond cystine accumulation. Am J Physiol Renal Physiol.

[45] Li M, Balamuthusamy S, Simon EE, Batuman V (2008). Silencing megalin and cubilin genes inhibits myeloma light chain endocytosis and ameliorates toxicity in human renal proximal tubule epithelial cells. Am J Physiol Renal Physiol.

[46] Hall AM, Bass P, Unwin RJ (2014). Drug-induced renal Fanconi syndrome. QJM.

[47] Schreiber A, Theilig F, Schweda F, Hocherl K (2012). Acute endotoxemia in mice induces downregulation of megalin and cubilin in the kidney. Kidney Int.

[48] Gu C, Wu L, Li X (2013). IL-17 family: cytokines, receptors and signaling. Cytokine.

[49] Kwok SK, Lee J, Yu D, Kang KY, Cho M, Kim HR (2015). A pathogenetic role for IL-21 in primary Sjogren syndrome. Nat Rev Rheumatol.

[50] Pitzalis C, Jones GW, Bombardieri M, Jones SA (2014). Ectopic lymphoid-like structures in infection, cancer and autoimmunity. Nat Rev Immunol.

[51] GeurtsvanKessel CH, Willart MA, Bergen IM, van Rijt LS, Muskens F, Elewaut D (2009). Dendritic cells are crucial for maintenance of tertiary lymphoid structures in the lung of influenza virus-infected mice. J Exp Med.

[52] Muniz LR, Pacer ME, Lira SA, Furtado GC (2011). A critical role for dendritic cells in the formation of lymphatic vessels within tertiary lymphoid structures. J Immunol.

[53] Veiga-Fernandes H, Coles MC, Foster KE, Patel A, Williams A, Natarajan D (2007). Tyrosine kinase receptor RET is a key regulator of Peyer's patch organogenesis. Nature.

[54] Peters A, Pitcher LA, Sullivan JM, Mitsdoerffer M, Acton SE, Franz B (2011). Th17 cells induce ectopic lymphoid follicles in central nervous system tissue inflammation. Immunity.

[55] Halle S, Dujardin HC, Bakocevic N, Fleige H, Danzer H, Willenzon S (2009). Induced bronchus-associated lymphoid tissue serves as a general priming site for T cells and is maintained by dendritic cells. J Exp Med.

[56] Sakai A, Sugawara Y, Kuroishi T, Sasano T, Sugawara S (2008). Identification of IL-18 and Th17 cells in salivary glands of patients with Sjogren's syndrome, and amplification of IL-17-mediated secretion of inflammatory cytokines from salivary gland cells by IL-18. J Immunol.

[57] Fei Y, Zhang W, Lin D, Wu C, Li M, Zhao Y (2014). Clinical parameter and Th17 related to lymphocytes infiltrating degree of labial salivary gland in primary Sjogren's syndrome. Clin Rheumatol.

[58] Chang A, Henderson SG, Brandt D, Liu N, Guttikonda R, Hsieh C (2011). In situ B cell-mediated immune responses and tubulointerstitial inflammation in human lupus nephritis. J Immunol.

[59] Rangel-Moreno J, Hartson L, Navarro C, Gaxiola M, Selman M, Randall TD (2006). Inducible bronchus-associated lymphoid tissue (iBALT) in patients with pulmonary complications of rheumatoid arthritis. J Clin Invest.

